# Association of dietary intake of polyphenols, lignans, and phytosterols with immune-stimulating microbiota and COVID-19 risk in a group of Polish men and women

**DOI:** 10.3389/fnut.2023.1241016

**Published:** 2023-08-03

**Authors:** Agnieszka Micek, Izabela Bolesławska, Paweł Jagielski, Kamil Konopka, Anna Waśkiewicz, Anna Maria Witkowska, Juliusz Przysławski, Justyna Godos

**Affiliations:** ^1^Statistical Laboratory, Jagiellonian University Medical College, Cracow, Poland; ^2^Department of Bromatology, Poznan University of Medical Sciences, Poznań, Poland; ^3^Department of Nutrition and Drug Research, Faculty of Health Sciences, Institute of Public Health, Jagiellonian University Medical College, Kraków, Poland; ^4^Department of Oncology, Jagiellonian University Medical College, Kraków, Poland; ^5^Department of Epidemiology, Cardiovascular Disease Prevention and Health Promotion, National Institute of Cardiology, Warszawa, Poland; ^6^Department of Food Biotechnology, Medical University of Bialystok, Białystok, Poland; ^7^Department of Biomedical and Biotechnological Sciences, University of Catania, Catania, Italy

**Keywords:** polyphenols, lignans, plant-sterols, phytochemicals, SARS-CoV-2, gut microbiota, nutrition

## Abstract

**Objectives:**

Devastating consequences of COVID-19 disease enhanced the role of promoting prevention-focused practices. Among targeted efforts, diet is regarded as one of the potential factors which can affect immune function and optimal nutrition is postulated as the method of augmentation of people’s viral resistance. As epidemiological evidence is scarce, the present study aimed to explore the association between dietary intake of total polyphenols, lignans and plant sterols and the abundance of immunomodulatory gut microbiota such as Enterococcus spp. and *Escherichia coli* and the risk of developing COVID-19 disease.

**Methods:**

Demographic data, dietary habits, physical activity as well as the composition of body and gut microbiota were analyzed in a sample of 95 young healthy individuals. Dietary polyphenol, lignan and plant sterol intakes have been retrieved based on the amount of food consumed by the participants, the phytochemical content was assessed in laboratory analysis and using available databases.

**Results:**

For all investigated polyphenols and phytosterols, except campesterol, every unit increase in the tertile of intake category was associated with a decrease in the odds of contracting COVID-19. The risk reduction ranged from several dozen percent to 70 %, depending on the individual plant-based chemical, and after controlling for basic covariates it was statistically significant for secoisolariciresinol (OR = 0.28, 95% CI: 0.11–0.61), total phytosterols (OR = 0.47, 95% CI: 0.22–0.95) and for stigmasterols (OR = 0.34, 95% CI: 0.14–0.72). We found an inverse association between increased β-sitosterol intake and phytosterols in total and the occurrence of *Escherichia coli* in stool samples outside reference values, with 72% (OR = 0.28, 95% CI: 0.08–0.86) and 66% (OR = 0.34, 95% CI: 0.10–1.08) reduced odds of abnormal level of bacteria for the highest compared with the lowest tertile of phytochemical consumption. Additionally, there was a trend of more frequent presence of Enterococcus spp. at relevant level in people with a higher intake of lariciresinol.

**Conclusion:**

The beneficial effects of polyphenols and phytosterols should be emphasized and these plant-based compounds should be regarded in the context of their utility as antiviral agents preventing influenza-type infections.

## 1. Introduction

The coronavirus 2019 (COVID-19) pandemic, caused by severe acute respiratory syndrome coronavirus 2 (SARS-CoV-2) and contributing to high morbidity and mortality in the last 3 years all over the world was a global challenge. The lack of satisfactory treatment against COVID-19, including therapeutic regimens or vaccines, and the urgent need of fighting the dangerous pathogen forced people to reach for alternatives. Until now, there is a concern about remedies that can stop the spread of microorganisms. Modifiable risk factors such as a proper diet abundant in vitamins and minerals, as well as in other constituents strengthening the natural immune system may be of primary importance in preventing influenza-type illnesses and minimizing their symptoms. A good nutritional status of individuals is mandatory to defeat the viruses and even might be treated as a measure of resilience toward pathogens such as SARS-CoV-2 (1). Various dietary components may shape the immune responses in different ways, among others, by determining the gut microbial composition. Specifically, the antioxidant constituents and anti-inflammatory agents of diet such as polyphenols and phytosterols have been shown to possess antiviral and immune-boosting properties. Notably, the evidence on health benefits of phytochemicals toward diseases underlined by oxidative stress and subclinical inflammation, including certain types of cancer (2, 3) and cardiovascular diseases (4, 5), has increased over the last few years. The research has demonstrated that dietary polyphenols can affect dendritic cells, increase the proliferation of B cells and T cells and might alter the phenotype of macrophages thus having an immunomodulatory effect (6). Phytosterols comprise many active compounds which determine their physiological functions, notably they have therapeutic potential against oxidative stress, gut dysbiosis, and inflammation (7). Plant-based diet rich in phytochemicals can help in lipid metabolism regulation counteracting virus entry into the cell and virus propagation (8). Therefore, there is growing evidence for recommending plant-based diets as an alternative effective and safe strategy which can prevent infections, although the research in a group of middle-aged, non-obese adults without comorbidities is limited. Moreover, SARS-CoV-2 as well as flu infections are easily disseminated in this group of subjects and also might pose a threat to them. While the elderly and those with underlying health conditions are at higher risk for severe complications from influenza-type and pneumonia-type diseases, young people can also experience dangerous health consequences as a result of these illnesses. The current burden of the disease highlighted the need for targeted efforts to decrease susceptibility to infectious illnesses, which in the European region during the 2022–2023 flu season were widespread and very severe. Although previous research explored how certain nutritional factors may affect COVID-19 infection *via* modulation of immune system (9, 10), to the best of our knowledge there is no study that investigated the potential of individual lignans and phytosterols in interacting with the immune system from the gastrointestinal tract, and in affecting viral infections, including SARS-CoV-2.Therefore, on the example of COVID-19, we aimed to perform the study examining the association of dietary intake of polyphenols and plant sterols with the abundance of immunomodulatory gut microbiota such as Enterococcus spp. and *Escherichia coli* and with the risk of contraction of the disease among physically active, non-obese early adults and early middle aged subjects without comorbidities. A better understanding of the protective dietary factors may help disseminate the strategies to counteract a variety of forthcoming viral infections.

## 2. Materials and methods

### 2.1. Study design, participants, and data collection

The present study was conducted in Poland and was designed to examine nutritional habits, physical activity and gastrointestinal microbiota of healthy young adults. The recruitment process was established through posting advertisements on social media and was further amplified by promotion requests, allowing to transmit the questionnaire to someone else. Details of the study have been described previously (11). Briefly, enrollment and data collection commenced in 2020. For each sex the separate arm of the study with the same research design was organized. The dates of examinations were between July 2020 and December 2020 for men and between October and November 2020 for women. Respondents were instructed not to change their daily routine, including eating habits and physical exercise patterns. During 1-week follow-up, participants were tracking their physical activity, total energy expenditure (TEE) and sleep duration using a Polar M430 watch and were keeping dietary records. After this time, stool samples were collected for gut microbiome testing, and core elements of anthropometry and body composition were recorded as well as participants were asked to complete a socio-demographic questionnaire. The diagnostics of the gastrointestinal microbiota was performed in the laboratory using KyberKompactPro test. The inclusion criteria covered: age between 25 and 45 years, body mass index (BMI) in the healthy weight range (18.5–24.9 kg/m^2^) or overweight range (25–29.9 kg/m^2^) and not having chronic diseases. Body weight and body composition were measured using Tanita’s Bioelectrical Impedance Analysis technology.

To check the hypothesis that a plant-based diet could be protective against the development of infections, we re-contacted all study participants in June 2021 and interviewed them regarding the prevalence and course of COVID-19 since the beginning of the pandemic. The questions included the information about duration of the illness, hospitalization and symptoms, and vaccination against the disease. Out of 104 individuals invited, nine were excluded because they did not have irrefutably confirmed diagnosis whether they had contracted COVID-19 (*n* = 4) or were vaccinated in too short interval of time since the examination (*n* = 5).

Finally, 95 persons were included in analyses among whom 24 had confirmed diagnosis of COVID-19 disease based on: positive PCR test results (*n* = 8), positive antibody test results (*n* = 7), typical COVID-19 symptoms, including loss of smell and taste (*n* = 9). The study was conducted in accordance with the Declaration of Helsinki for medical research and obtained positive approval from the Bioethics Committee of Jagiellonian University (No. 1072.6120.5.2020 and 1072.6120.202.2019). Following a careful explanation of research conditions and procedures, an informed consent was signed by all subjects before they participated in the study.

### 2.2. Dietary assessment and other measurements

Based on the data collected in 7-day diaries, the nutritional value of foods was determined using the Dieta 6.0 program developed by the National Food and Nutrition Institute in Poland and including information on total fat and individual fatty acids, protein and individual amino acids, carbohydrates, cholesterol, fiber, vitamins and minerals. The method of determination of polyphenols and phytosterols content in foods was previously described in detail (12–14). Dietary polyphenol and plant sterol intakes were calculated according to the amount of various kinds of foods and dishes consumed by the participants combined with their phytochemical content. Total polyphenol content was assessed mostly in laboratory analysis based on 367 foods and dishes consumed typically in Poland and taking into consideration the degree of processing with the division to uncooked/raw products and products submitted to culinary treatments (13). Additionally, the available databases were searched to retrieve the mean content of lignans (3 data sources, primary Dutch lignan database) and plant sterols (13 data sources, e.g., British database of Food Composition, the USDA database) in all foods, as well as total polyphenols (Phenol-Explorer database) for a very small number of foods not subjected to laboratory analysis (12–14). Product-specific macro-, micronutrient, polyphenol, lignan and plant sterol intake was obtained as a result of the multiplication of their content in food by the daily consumption of each food. Finally, these values were summed across all foods which the individual subject consumed. To reduce extraneous variation and eliminate noncausal association with disease due to confounding generated by the correlation of total energy intake with both nutrients intake and the disease risk, daily consumption of each phytochemical was additionally adjusted for total energy intake using the residual method (15). The categorization of phytochemical intakes was based on tertile distribution. Regarding covariates used in the analysis, total energy intake [kcal], BMI [kg/m^2^], age [years], and physical activity [hours per day] (logarithmically transformed) were incorporated into the models as continuous variables. Based on the body fat (BF) percentage and cutoff points by age and sex suggested by Gallagher et al. (16), the subjects were divided into the following groups: underfat, normal fat and overfat. Alcohol consumption was categorized as (i) none or moderate when consuming less than 5 g ethanol per day for women and less than 10 g/d for men, and (ii) regular otherwise. Sex, diet and smoking status were dichotomized as male/female, traditional/vegetarian and current/other, respectively.

### 2.3. Statistical analysis

Categorical variables were depicted by absolute numbers and percentages whereas continuous features were described using means and standard deviations. Background characteristics of individuals were made for different tertile categories of total lignan and total phytosterol intake, and subsequently were compared across premade groups. To reduce the right skewness of daily phytochemical intakes and physical activity, logarithmic transformation (using base 2) was adopted. Significant improvements in the shape of their distribution to forms closely resembling the Gaussian curve were noted for all variables. Therefore, Log 2 transformed phytochemical intakes were compared between participants who have contracted and who have not contracted COVID-19 disease applying Student *T*-test. Differences between tertile categories of consumptions in univariable analyses were checked with Chi-square or Fisher exact test in the case of categorical variables, and with ANOVA in the case of continuous variables (after checking normality assumption and homogeneity of variance). The odds ratios (ORs) and 95% confidence intervals (CIs) were retrieved from multivariable logistic regression models after controlling for (i) age, total energy intake (model 1); (ii) additionally for sex, diet, body fat and smoking status (model 2); and finally for (iii) covariates in model 2 and BMI, physical activity and alcohol consumption (model 3). Different levels of adjustment allowed for verification whether associations were independent of the aforementioned variables, ensuring the robustness and stability of the findings. The logistic regression analysis was modeled by introducing exposures as: (i) three-level categorical variables with lowest tertiles as referent categories, (ii) as continuous variables (logarithmically transformed) to show the effects associated with a double increment of intake (per 1 unit increase in log 2), and additionally as (iii) score variables which were constructed by coding tertile groups with 1, 2 and 3 and treated as numerical. *p*-values from two-sided tests were reported under a significance level of 0.05. R software (Development Core Team, Vienna, Austria, version 4.0.4) was used for all the statistical analysis. Additionally, the *post hoc* power analysis based on the multiple logistic regression was performed applying G*Power tool (version 3.1). We verified the hypothesis that increasing total polyphenols intake for 1 unit in log2 scale significantly changes the chance of having contracted COVID-19. We set: 95 subjects, OR = 0.20, probability of COVID-19 incidence when adequate amounts of phytochemicals were consumed: 0.16, variability in the main exposure that is accounted for by other covariates: 0.25. By default, two-tailed test and probability of type I error at a level 0.05 have been maintained. We obtained a highly satisfactory power equal to 0.995.

## 3. Results

### 3.1. Baseline characteristics of participants

The study was conducted on 95 persons, 73 men (76.8%) and 22 women (23.2%), aged 25–45 years (mean = 34.66, SD = 5.76), every fourth of whom (*n* = 24) have contracted COVID-19. Participants from various tertiles of lignans and phytosterols did not differ in age, sex, marital status, BMI categories, body fat, smoking status, physical activity, total energy intake, total energy expenditure and sleep duration. However, there were significantly more vegetarians and regular alcohol drinkers in the highest category of phytosterols consumers compared with others. Detailed characteristics of examined adults are presented in Table 1.

**Table 1:** Baseline characteristics of the study participants (*N* = 95).

	Total lignan intake			Total phytosterol intake		
Variable	T1 (*N* = 32)	T2 (*N* = 31)	T3 (*N* = 32)	T1 (*N* = 32)	T2 (*N* = 31)	T3 (*N* = 32)
Age [years], mean (sd)	34.53 (6.15)	33.68 (5.68)	35.75 (5.42)	35.81 (6.49)	34.61 (5.44)	33.56 (5.22)
Sex, *n* (%)						
Male	24 (75.00)	26 (83.87)	23 (71.88)	24 (75.00)	23 (74.19)	26 (81.25)
Female	8 (25.00)	5 (16.13)	9 (28.13)	8 (25.00)	8 (25.81)	6 (18.75)
Marital status, *n* (%)						
Single or divorced	18 (56.25)	16 (51.61)	14 (43.75)	13 (40.63)	16 (51.61)	19 (59.38)
Married or cohabiting	14 (43.75)	15 (48.39)	18 (56.25)	19 (59.38)	15 (48.39)	13 (40.63)
BMI category, *n* (%)						
Normal	20 (62.50)	22 (70.97)	24 (75.00)	22 (68.75)	20 (64.52)	24 (75.00)
Overweight	12 (37.50)	9 (29.03)	8 (25.00)	10 (31.25)	11 (35.48)	8 (25.00)
Diet, *n* (%)						
Traditional	22 (68.75)	16 (51.61)	16 (50.00)	23 (71.88)	23 (74.19)	8 (25.00)***
Vegetarian	10 (31.25)	15 (48.39)	16 (50.00)	9 (28.13)	8 (25.81)	24 (75.00)
Body fat, *n* (%)						
Under fat	2 (6.25)	4 (12.90)	4 (12.50)	4 (12.50)	3 (9.68)	3 (9.38)
Normal	21 (65.63)	25 (80.65)	23 (71.88)	20 (62.50)	23 (74.19)	26 (81.25)
Overfat	9 (28.13)	2 (6.45)	5 (15.63)	8 (25.00)	5 (16.13)	3 (9.38)
Smoking, *n* (%)						
No	28 (87.50)	27 (87.10)	28 (87.50)	28 (87.50)	28 (90.32)	27 (84.38)
Yes	4 (12.50)	4 (12.90)	4 (12.50)	4 (12.50)	3 (9.68)	5 (15.63)
Alcohol, *n* (%)						
None or moderate	21 (65.63)	21 (67.74)	18 (56.25)	23 (71.88)	23 (74.19)	14 (43.75)*
Regular	11 (34.38)	10 (32.26)	14 (43.75)	9 (28.13)	8 (25.81)	18 (56.25)
Energy intake [kcal], mean(sd)	2,261 (575)	2,221 (503)	2,174 (394)	2,133 (445)	2,364 (611)	2,164 (379)
BMI [kg/m^2^], mean (sd)	23.65 (2.69)	23.43 (2.60)	22.93 (2.69)	23.65 (2.46)	23.50 (2.89)	22.86 (2.60)
Log Physical Activity [h/d], mean (sd)	0.58 (0.14)	0.56 (0.15)	0.58 (0.19)	0.54 (0.12)	0.61 (0.17)	0.56 (0.18)
TEE [kcal], mean (sd)	2,529 (461)	2,590 (385)	2,523 (491)	2,486 (410)	2,622 (466)	2,536 (461)
Sleep duration [h], mean (sd)	7.44 (0.70)	7.44 (0.91)	7.44 (0.86)	7.44 (0.84)	7.44 (0.84)	7.44 (0.84)

Results are expressed as *n* (%), or mean (sd), T1, T2, T3, tertile groups; TEE, total energy expenditure; BMI, body mass index, mean, *p* values are based on Chi-squared test of independence or ANOVA test, *p < 0.05, **p < 0.01, ***p < 0.001.

### 3.2. Relationship of lignan and phytosterol intake with COVID-19 contraction

**Figure 1:**
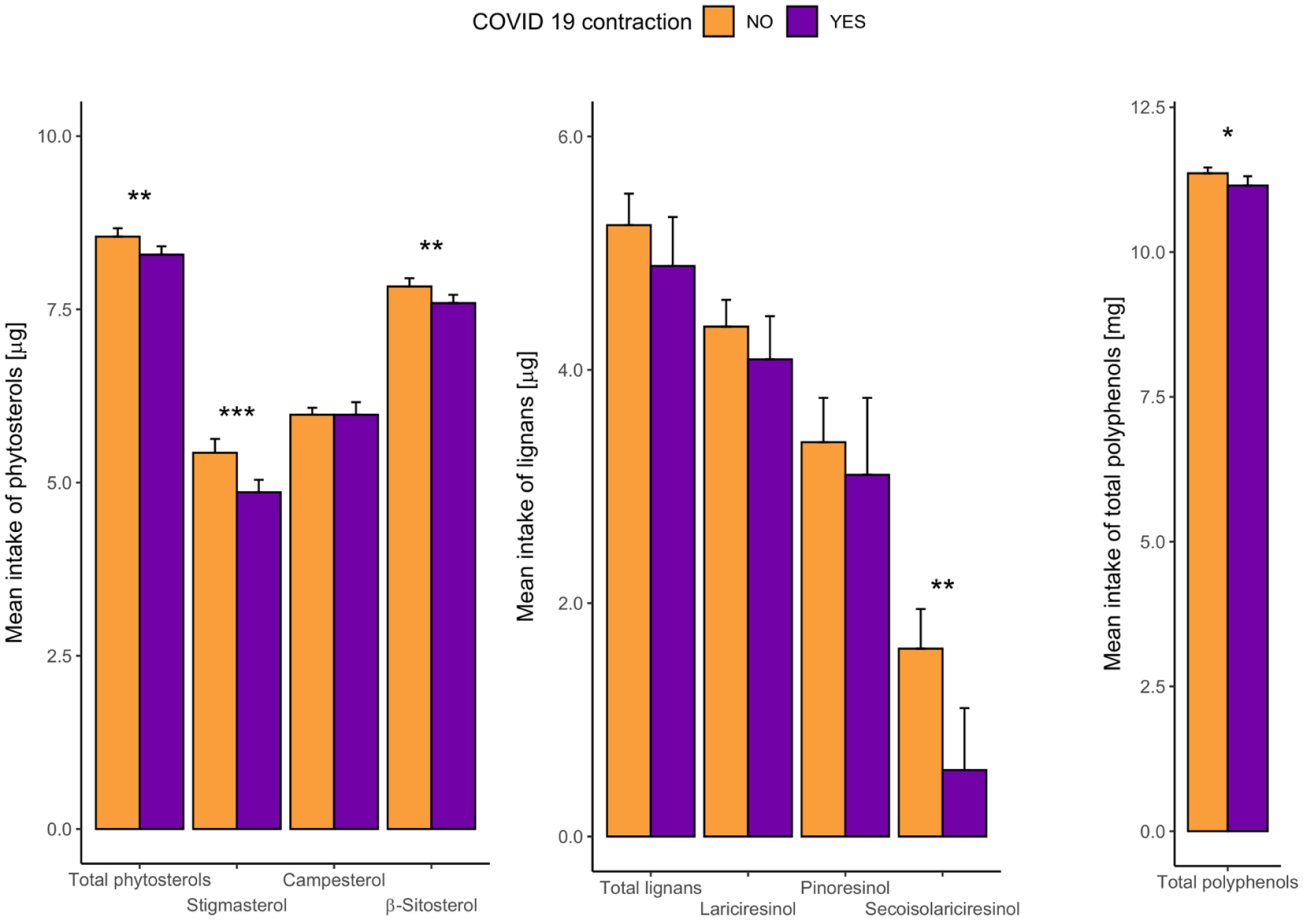
Comparison of distribution of energy adjusted logarithmically transformed daily consumption of specific phytochemicals between respondents who have contracted and who have not contracted COVID-19. For better visualization particular phytochemical intake were expressed in different units. *p < 0.05, **p < 0.01, ***p < 0.001 for Student *T*-test analysis (*N* = 95).

Intake of total polyphenols, and total and major groups of lignans and phytosterols by categories of people who have contracted and who have not contracted COVID-19 was shown in Figure 1; Supplementary Table S1. Habitual consumption of total polyphenols, secoisolariciresinol, total phytosterols, stigmasterol and β-sitosterol was significantly lower among those who fell ill with COVID-19.

To better visualize the relationship between a phytochemical-rich diet and the prevalence of the disease, percentages of cases across tertiles of exposures, as well as odds ratios for 1 category increase in tertile of intake and for doubling intake were depicted in Table 2. In univariable analysis, both chi-squared test and logistic regression models have shown that higher consumption of secoisolariciresinol, total phytosterols and stigmasterol was associated with lower risk of COVID-19. Additionally, compared with lower tertiles, a significantly lower frequency of contraction of COVID-19 was noted in the highest tertile of consumption of matairesinol and decreased chance of the illness was observed for people with greater intake of total polyphenols and β-sitosterol (Table 2).

**Table 2:** Association between phytochemical intake and COVID-19 contraction – comparison of distribution of risk of disease by tertiles of consumptions and crude logistic regression analysis for continuous exposure level (*N* = 95).

Phytochemicals	Phytochemical-specific tertiles			OR (95% CI)	
	T1 (*N* = 32)	T2 (*N* = 31)	T3 (*N* = 32)	Per 1 category of tertile increase	Per 1 unit increase in Log2
	COVID-19 contraction, *n* (%)				
Total polyphenols [mg]	11 (34.4)	8 (25.8)	5 (15.6)	0.60 (0.33–1.08)	0.29 (0.09–0.92)*
Total lignans [μg]	11 (34.4)	6 (19.4)	7 (21.9)	0.72 (0.40–1.27)	0.74 (0.47–1.15)
Lariciresinol [μg]	10 (31.3)	7 (22.6)	7 (21.9)	0.78 (0.44–1.38)	0.74 (0.44–1.23)
Matairesinol [ng]	9 (28.1)	12 (38.7)	3 (9.4)*	0.60 (0.33–1.08)	0.90 (0.70–1.16)
Pinoresinol [μg]	8 (25.0)	9 (29.0)	7 (21.9)	0.92 (0.52–1.62)	0.90 (0.67–1.20)
Secoisolariciresinol [μg]	15 (46.9)	6 (19.4)	3 (9.4)**	0.33 (0.17–0.64)**	0.50 (0.31–0.81)**
Total phytosterols [mg]	12 (37.5)	9 (29.0)	3 (9.4)*	0.45 (0.24–0.84)*	0.21 (0.05–0.84)*
Stigmasterol [mg]	14 (43.8)	7 (22.6)	3 (9.4)**	0.37 (0.19–0.70)**	0.26 (0.11–0.64)**
Campesterol [mg]	8 (25.0)	10 (32.3)	6 (18.8)	0.85 (0.48–1.49)	1.03 (0.35–3.03)
β-sitosterol [mg]	12 (37.5)	8 (25.8)	4 (12.5)	0.50 (0.27–0.92)*	0.23 (0.06–0.89)*

Results are expressed as *n* (%), or OR (95% CI), OR, odds ratio; CI, confidence interval; T1, T2, T3, tertile groups; *p < 0.05, **p < 0.01, ***p < 0.001 from Chi-squared test or logistic regression analysis.

**Figure 2:**
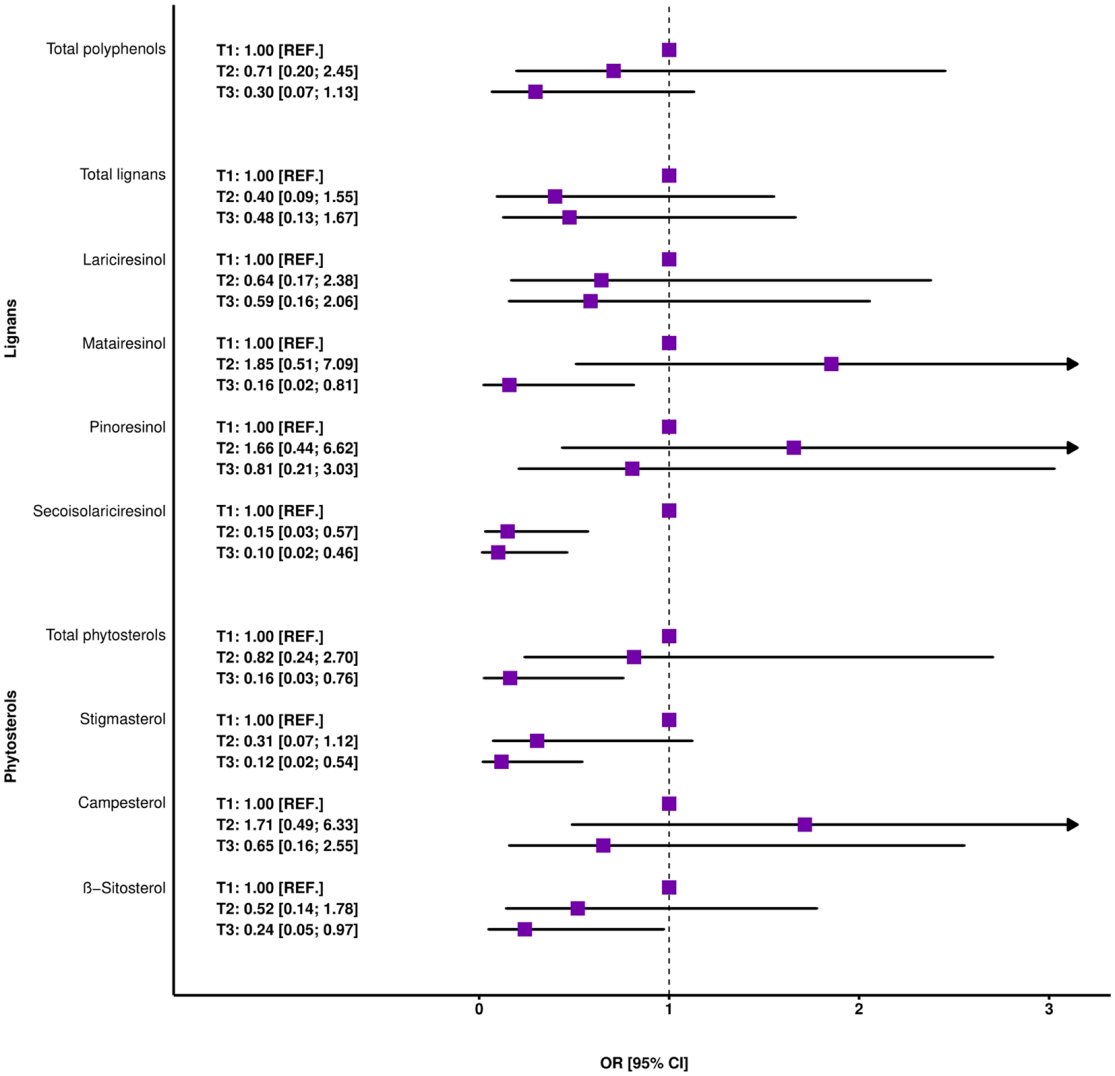
Odds ratios of COVID-19 contraction across specific-phytochemical tertiles. The results of fully adjusted models after controlling for age, total energy intake, sex, diet, smoking status, BF, BMI, physical activity, and alcohol consumption (*N* = 95).

The negative associations between COVID-19 prevalence and dietary intake of secoisolariciresinol, total phytosterols and stigmasterol were confirmed in multivariable analysis, showing very stable and robust results after controlling for different sets of potential confounders. In fully adjusted models, independently of age, total energy intake, sex, diet, smoking status, BF, BMI, physical activity and alcohol consumption, the diet richest in specific phytochemical diminished the odds of the occurrence of COVID-19 about 90, 84, and 88% compared with the diet poorest in these compounds (OR = 0.10, 95% CI: 0.02–0.46 for secoisolariciresinol, OR = 0.16, 95% CI: 0.03–0.76 for total phytosterols and OR = 0.12, 95% CI: 0.02–0.54 for stigmasterol, Table 3; Figure 2). These relations were also reflected by a decline in the risk of the disease with each movement to a higher category of tertile intake (OR = 0.28, 95% CI: 0.11–0.61 for secoisolariciresinol, OR = 0.47, 95% CI: 0.22–0.95 for phytosterols and OR = 0.34, 95% CI: 0.14–0.72 for stigmasterols) and with doubling of intake (OR = 0.47, 95% CI: 0.24–0.79 for secoisolariciresinol, OR = 0.23, 95% CI: 0.04–1.01 (marginally significant) for phytosterols and OR = 0.29, 95% CI: 0.09–0.72 for stigmasterol, Table 3). Moreover, the evidence of protective effect against COVID-19 contraction was found for total polyphenols, matairesinol and β-sitosterol, in most cases with significant or marginally significant results and a reduction in the odds of the disease ranging from 70 to 84% in analysis comparing extreme categories of intake, from 44 to 51% in analysis reflecting change category of tertile to one level higher and, except matairesinol, from 74 to 80% when doubling intake, although wide confidence intervals were observed (Table 3; Figure 2). No univocal patterns of trends could have been found concerning total lignans, two individual lignan groups, namely lariciresinol and pinoresinol and one individual phytosterol group, namely campesterol; despite a general tendency of decreasing the risk of COVID-19 observed with a higher intake, no result reached statistical significance (Table 3).

**Table 3:** Association between phytochemical intake and COVID-19 contraction.

Phytochemicals	T1 (*N* = 31)	T2 (*N* = 31)	T3 (*N* = 32)	Per 1 category of tertile increase	Per 1 unit increase in Log2
Total polyphenols [mg]					
Model 1	1 (ref.)	0.78 (0.24–2.52)	0.28 (0.07–0.96)*	0.55 (0.29–1.00)	0.22 (0.06–0.74)*
Model 2	1 (ref.)	0.70 (0.20–2.41)	0.30 (0.07–1.09)	0.56 (0.28–1.06)	0.21 (0.05–0.76)*
Model 3	1 (ref.)	0.71 (0.20–2.45)	0.30 (0.07–1.13)	0.56 (0.27–1.07)	0.20 (0.05–0.77)*
Total lignans [μg]					
Model 1	1 (ref.)	0.44 (0.12–1.47)	0.43 (0.12–1.36)	0.64 (0.34–1.17)	0.70 (0.43–1.10)
Model 2	1 (ref.)	0.44 (0.11–1.63)	0.47 (0.12–1.62)	0.68 (0.35–1.29)	0.73 (0.44–1.17)
Model 3	1 (ref.)	0.40 (0.09–1.55)	0.48 (0.13–1.67)	0.69 (0.35–1.30)	0.74 (0.44–1.17)
Lariciresinol [μg]					
Model 1	1 (ref.)	0.62 (0.18–2.03)	0.52 (0.15–1.71)	0.72 (0.39–1.31)	0.66 (0.37–1.11)
Model 2	1 (ref.)	0.68 (0.19–2.38)	0.58 (0.16–2.00)	0.76 (0.40–1.42)	0.70 (0.38–1.20)
Model 3	1 (ref.)	0.64 (0.17–2.38)	0.59 (0.16–2.06)	0.76 (0.40–1.44)	0.69 (0.37–1.20)
Matairesinol [ng]					
Model 1	1 (ref.)	1.41 (0.46–4.43)	0.24 (0.05–0.97)*	0.56 (0.29–1.04)	0.89 (0.67–1.17)
Model 2	1 (ref.)	1.68 (0.47–6.20)	0.20 (0.03–0.97)*	0.51 (0.24–1.03)	0.90 (0.65–1.21)
Model 3	1 (ref.)	1.85 (0.51–7.09)	0.16 (0.02–0.81)*	0.49 (0.23–1.01)	0.88 (0.63–1.21)
Pinoresinol [μg]					
Model 1	1 (ref.)	1.29 (0.39–4.34)	0.76 (0.22–2.59)	0.88 (0.48–1.58)	0.88 (0.65–1.18)
Model 2	1 (ref.)	1.66 (0.46–6.25)	0.80 (0.21–2.98)	0.89 (0.47–1.67)	0.90 (0.66–1.22)
Model 3	1 (ref.)	1.66 (0.44–6.62)	0.81 (0.21–3.03)	0.88 (0.46–1.67)	0.90 (0.65–1.22)
Secoisolariciresinol [μg]					
Model 1	1 (ref.)	0.18 (0.05–0.61)**	0.09 (0.02–0.37)**	0.28 (0.12–0.57)**	0.47 (0.26–0.75)**
Model 2	1 (ref.)	0.18 (0.05–0.63)*	0.11 (0.02–0.46)**	0.29 (0.12–0.62)**	0.48 (0.26–0.79)**
Model 3	1 (ref.)	0.15 (0.03–0.57)**	0.10 (0.02–0.46)**	0.28 (0.11–0.61)**	0.47 (0.24–0.79)*
Total phytosterols [mg]					
Model 1	1 (ref.)	0.88 (0.28–2.74)	0.19 (0.04–0.73)*	0.49 (0.25–0.90)*	0.25 (0.05–0.88)*
Model 2	1 (ref.)	0.81 (0.25–2.61)	0.16 (0.03–0.72)*	0.47 (0.22–0.92)*	0.23 (0.04–0.99)*
Model 3	1 (ref.)	0.82 (0.24–2.70)	0.16 (0.03–0.76)*	0.47 (0.22–0.95)*	0.23 (0.04–1.01)
Stigmasterol [mg]					
Model 1	1 (ref.)	0.43 (0.13–1.36)	0.14 (0.03–0.54)**	0.39 (0.19–0.74)**	0.30 (0.11–0.69)**
Model 2	1 (ref.)	0.32 (0.09–1.11)	0.13 (0.02–0.56)*	0.35 (0.16–0.73)**	0.29 (0.10–0.71)*
Model 3	1 (ref.)	0.31 (0.07–1.12)	0.12 (0.02–0.54)**	0.34 (0.14–0.72)**	0.29 (0.09–0.72)*
Campesterol [mg]					
Model 1	1 (ref.)	1.87 (0.58–6.37)	0.68 (0.19–2.41)	0.85 (0.46–1.52)	1.20 (0.38–3.64)
Model 2	1 (ref.)	1.63 (0.48–5.73)	0.60 (0.16–2.19)	0.80 (0.42–1.47)	1.13 (0.34–3.63)
Model 3	1 (ref.)	1.71 (0.49–6.33)	0.65 (0.16–2.55)	0.83 (0.42–1.58)	1.19 (0.35–3.88)
β-sitosterol [mg]					
Model 1	1 (ref.)	0.59 (0.18–1.85)	0.23 (0.05–0.81)*	0.49 (0.25–0.91)*	0.24 (0.05–0.87)*
Model 2	1 (ref.)	0.52 (0.15–1.72)	0.24 (0.05–0.93)*	0.49 (0.24–0.96)*	0.24 (0.04–1.02)
Model 3	1 (ref.)	0.52 (0.14–1.78)	0.24 (0.05–0.97)*	0.49 (0.23–0.98)*	0.24 (0.04–1.02)

Results of multiple logistic regression analysis for tertiles of consumption and for continuous exposure (*N* = 95). Results are presented as ORs (95% CIs), T1, T2, T3, tertile groups, *p < 0.05, **p < 0.01, ***p < 0.001 from multiple logistic regression analysis, Model 1, adjusted for age and total energy intake; Model 2, adjusted for variables in model 1 and sex, diet, smoking status, BF; Model 2, adjusted for variables in model 2 and for BMI, physical activity and alcohol consumption (*N* = 95).

### 3.3. Relationship of lignan and phytosterol intake with the immunostimulatory microbiota *Escherichia coli* and Enterococcus spp. as well as with immunomodulatory profile of the diet assessed by POLA index

**Figure 3:**
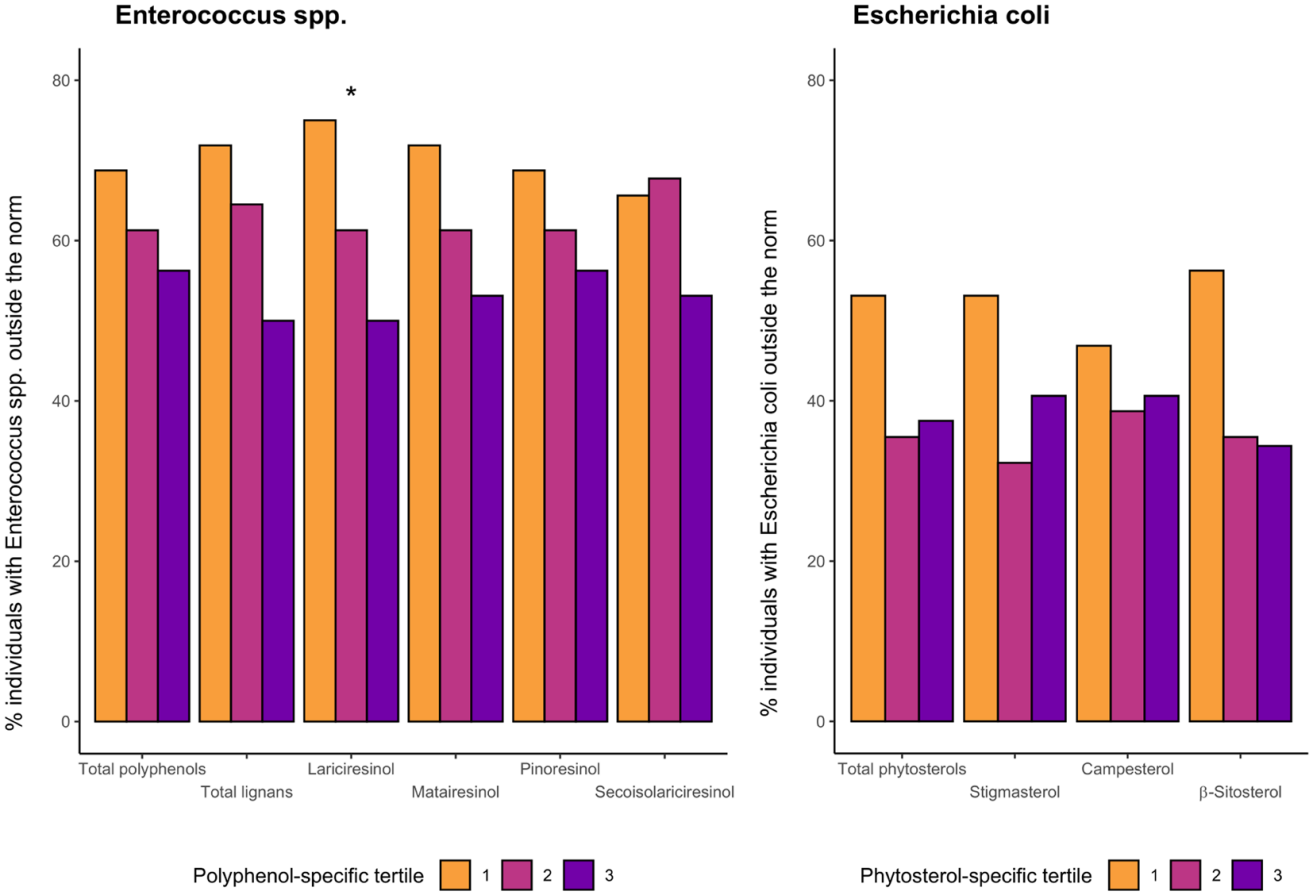
Comparison of percentage of people in whom the amount of bacteria Enterococcus spp. and *Escherichia coli* was outside the norm across specific-polyphenol tertiles, *p < 0.05, **p < 0.01, ***p < 0.001 for logistic regression analysis testing decreasing trend between tertiles after controlling for age, sex, diet, and total energy intake (*N* = 95).

**Figure 4:**
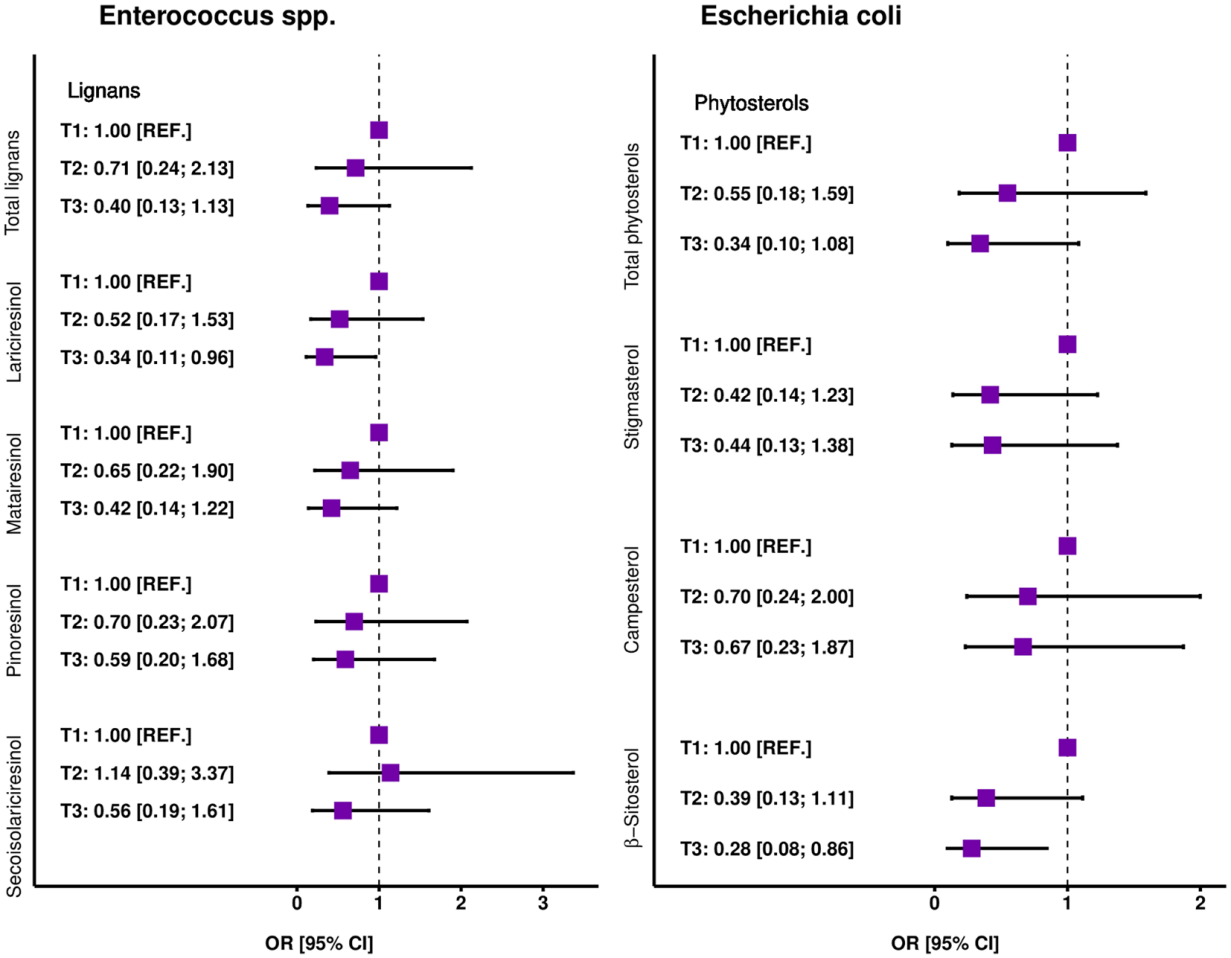
Odds ratios of bacteria Enterococcus spp. and *Escherichia coli* outside the norm across specific-polyphenol tertiles. Multiple logistic regression after controlling for age, sex, diet, and total energy intake (*N* = 95).

The frequencies and odds ratios of the occurrence of abnormal amounts of each of the two strains of immune-stimulating microbiota (below 10^6^ CFU/g in feces) within groups of people with different consumption of phytochemicals was shown in Figures 3, 4. Generally, a decreasing trend in the occurrence of abnormal values of Enterococcus spp. was noted across tertile categories of total polyphenols and all types of lignans except secoisolariciresinol. In the case of *Escherichia coli* a tendency of analogical trends was observed between premade groups of phytosterols. However, the result for Enterococcus spp. was statistically significant only for lariciresinol (*p* < 0.05) and on the boundary of significance only for total lignans (*p* < 0.1), showing lowering the odds of abnormal values about 42 and 38%, respectively, with each increment of tertile category. The largest disparities in the frequency of prevalence of aberrant values of strains of *Escherichia coli* were found between tertiles of β-sitosterols intake. Independently of age, sex, diet, and total energy intake, persons in the third tertile of β-sitosterols compared with the first tertile had 72% reduced odds of having an abnormal level of bacteria compared with those from the first tertile, despite a very wide confidence interval (OR = 0.28, 95% CI: 0.08–0.86). No other statistically significant results were observed (Figures 3, 4; Supplementary Table S2).

Moreover, there was a positive association between a higher intake of lignans (however nonsignificant) and phytosterols and more beneficial immunomodulatory profile of the diet assessed by the POLA index which has been previously shown to be negatively related to the risk of COVID-19 (Supplementary Table S3) (17).

## 4. Discussion

The present study examined the relationship between phytochemical consumption, gut microbiota and the risk of COVID-19 disease among non-obese 25-45-year-old subjects without comorbidities. The results showed that higher intake of total polyphenols, specific lignans such as secoisolariciresinol and matairesinol, as well as total phytosterols and some subclasses: stigmasterols and β-sitosterols was associated with a lower risk of COVID-19.

There are currently no human studies available evaluating the effectiveness of higher dietary polyphenol, lignan and phytosterol intake in reducing COVID-19 risk. However, the antiviral efficacy of polyphenols including lignans and plant sterols has been confirmed against SARS-CoV, MERS-CoV, Ebola virus, HIV, influenza virus and other viruses causing respiratory tract infections (18–21). Recent studies have also demonstrated their beneficial effects against the SARS-CoV-2 virus (18, 22–24) resulting from their ability to bind to peak protein sites on the ACE2 receptor used by SARS-CoV-2 to infect cells (25, 26), regulate ACE2 expression and also interfere with SARS-CoV-2 replication by inhibiting the virus protease or inhibiting SARS-CoV-2 RNA-dependent RNA polymerase (RdRp) (27, 28). The antiviral activity of some of them is comparable to or stronger than pharmacological agents (naringenin, vs. remdesivir (29), citrus flavonoids and polyphenols from Curcuma spp. vs. lopinavir and nafamostat (30)).

Although diet alone is not sufficient for the prevention of any virus infection, adequate nutrition is viewed as one of the best complementary approaches for controlling many types of infections, including SARS-CoV-2. Optimal dietary intake of macro- and micronutrients and other bioactive constituents may affect the immune system and thus strengthen the protection against influenza-like illnesses such as COVID-19 disease (1, 11, 17, 31). Unfortunately, the pandemic has had a significant impact on nutritional habits, yet dietary changes have manifested differently and to various extents among men and women as well as in people with distinct socioeconomic status (32–34). For example, improvements in line with a Mediterranean diet which is proven to help boost immunity, were associated with higher education, wealth, skilled manual occupations and male sex (33). Nevertheless, the transition to the new habits during the pandemic, primarily, was associated with negative eating behaviors.

It has been reported that consumptions of polyphenols and phytosterols such as β-sitosterol, campesterol, and stigmasterol, are beneficial for the human immune system and health due to their antioxidant, anti-inflammatory and cholesterol-lowering activity (7, 35, 36). Also, lignans that include secoisolariciresinol and matairesinol are among the most promising immunotherapeutics (37) that exhibit antioxidant and anti-inflammatory activity (38). The immune system is critical for the clearance of a variety of infections. In the case of COVID-19, induced cytokine release syndrome is suggested to play a pivotal role in the pathology of the disease. Therefore, there is a significant concern regarding the search for methods of treatment that focus on prevention of cytokine storm in ill patients. Such an activity is demonstrated by polyphenols and phytosterols, which, through their effects on macrophages, inhibit the secretion of the pro-inflammatory mediators interleukin-1-beta (IL-1ß), IL-2, IL-6, γ-interferon (IFN-γ), and tumor necrosis factor α (TNF-α) reducing inflammation caused by hyperactivation of cytokines (39–41). Anti-inflammatory and preventive effects against diseases associated with immune dysregulation are shown, for example, by polyphenols in red wine (they raise interleukin IL-21 levels and reduce the release of IL-1β and IL-6) (42) or polyphenols from green tea, pomegranate, grape seed, mango (6).

The high capacity to reduce oxidative stress and overproduction of reactive oxygen species (ROS) exacerbates the anti-inflammatory effects of polyphenols and phytosterols (43–45). Polyphenols can also enhance resistance to foreign pathogens through other inflammation-related pathways like activation of T regulatory cells (Treg), which can suppress cytotoxic T cell function (6, 46), affecting dendritic cells, increasing B and T lymphocyte proliferation and inducing apoptosis (6). On the other hand, some phytosterol compounds, in addition to their ability to attenuate the inflammatory response in lipopolysaccharide-induced macrophage models (47), may also exert antiproliferative effects (β-sitosterol) (48).

Both polyphenols and phytosterols reduce cholesterol levels in the cell membrane or destabilize the structure of lipid rafts, which are the main docking sites for COVID-19 entry and genome release (49). Cholesterol is also essential for the replication and infectivity of enveloped viral particles, and influences the molecular and cellular events of immune cells and subsequent biological responses through many other mechanisms (50). Plant sterols have similar chemical structure to cholesterol, and esters of both compounds can be seen as the rivals to each other due to their competition for hydrolysis by enzymes in the gastrointestinal tract. As phytosterols are more lipophilic, they can contribute to the reduction of micellar solubility of cholesterol in the intestine and are capable of eliciting the reduction of the cholesterol absorption by enterocytes (36). Phytosterols (sitosterol and campesterol) can activate the bile acid excretion pathway and accelerate cholesterol metabolism (51–53), reduce cholesterol synthesis and disrupt cholesterol homeostasis (stigmasterol) (54, 55). A therapy with a polyphenol composition resulted in a significant increase in HDL fraction cholesterol (56), low levels of which were associated with a more severe course of COVID-19 (57). The cardioprotective effects of these compounds (58, 59) may also improve the course of COVID-19 in patients with cardiovascular disease. They also have anti-diabetic effects (43, 60, 61) and may reduce obesity (62).

Numerous studies confirm that a diet high in plant-based products containing, among others, phytosterols naturally present in the cell membranes of lipid-rich plants (nuts, seeds, legumes, olive oil (63)), is associated with a lower risk of infection and a milder course of COVID-19 (64, 65). Some dietary patterns including products abundant in polyphenols and phytosterols have been also shown to be competent to antagonize inflammation by many pathways. The Mediterranean diet characterized by a high amount of antioxidant vitamins and phytochemicals has been reported to reduce oxidative stress, block pro-inflammatory cytokines, suppress inflammatory and increase antioxidant gene expression, as well as activate transcription factors that counteract chronic inflammation (66, 67). In the large cohort of the MOLI-SANI study, a Mediterranean eating pattern as well as specifically flavonoid and lignan intakes measured by Polyphenol Antioxidant Content (PAC) score, have been proven to be related to novel cellular biomarkers of low-grade inflammation such as platelet, leucocyte counts and granulocyte:lymphocyte ratio (68). Regarding the possible effect of other dietary, clinical and environmental factors, the flavonoid and lignan content of diet explained a relatively high proportion of the variation of INFLA score evaluating the synergetic effect of inflammatory biomarkers in both men and women (66).

Interestingly, in our study among the subjects who consumed the most phytosterols there were regular alcohol drinkers. This was related to their consumption of beer, which was a source of a significant amount of these compounds (69, 70). Although the amount of phytosterols in beer is considered too low for a health effect, some wheat beers with a high yeast content, or with the addition of whole grain, may contain higher levels of ergosterol or sitosterol (70). The benefits of alcohol consumption remain controversial concerning both the type of alcohol and the drinking pattern, and it is difficult to recommend alcohol as a source of phytosterols.

Our study shows that while habitual intake of the analyzed compounds did not protect against COVID-19, higher intake of total polyphenols, specific lignans such as secoisolariciresinol, total phytosterols and some subclasses: stigmasterol and β-sitosterol was associated with lower risk of COVID-19. Also in other studies, high doses of polyphenols (71) and a high intake of β-sitosterol (72) have been shown to possess a protective effect against COVID-19. In an *in silico* computational study secoisolariciresinol was found to be more effective against non-structural proteins (nsp10 and nsp16) of SARS-CoV-2 than remdesivir and more significant than lopinavir (73). There are no results to date on the effect of an increased matairesinol intake in COVID-19. A diet with a significant amount of matairesinol compared to a typical northern Italian diet resulted in reduced vascular inflammation and endothelial dysfunction (74). Matairesinol attenuated sepsis-induced brain damage (45). However, the beneficial role of matairesinol in reducing the frequency of COVID-19 contractions was demonstrated here for the first time.

Various factors influence the incidence and course of COVID-19. COVID-19 infection has been shown to have the lowest mortality rate among children with a log-linear increase among the elderly (75), sexual dimorphism with a tendency toward greater severity and mortality among men (76), a worse prognosis and higher mortality among those who are obese (77) or consume large amounts of alcohol (78). In contrast, people with a balanced diet, who were physically active and did not use stimulants were at significantly lower risk of COVID-19 infection (11). However, our study showed that regardless of age, total energy intake, sex, diet, smoking status, BF, BMI, physical activity and alcohol consumption, a diet richest in secoisolariciresinol, total phytosterols and stigmasterol reduced the odds of COVID-19 by 90, 84 and 88% respectively, and a diet richest in total polyphenols, matairesinol and β-sitosterol showed a protective effect against COVID-19 contraction with reduced odds of the disease ranging from 70 to 84% in analysis comparing extreme categories of intake. These relationships were also reflected in a decrease in the risk of disease with each shift to a higher category of tertile intake and with doubling of intake for most of them. The implication is that sufficiently high dietary intake of these phytochemicals may affect the infection rate, course and mortality caused by SARS-CoV-2 more strongly than the other factors. Their efficacy against COVID-19 when consumed with the diet was independent of low bioavailability (79, 80), environmental factors and enzymatic activity in the gastrointestinal tract, which are indicated as factors that potentially reduce the concentration of polyphenols and even cause partial or complete loss of their bioactivity (79). Thus, it appears that the pharmacological and molecular effects of dietary phytochemicals may be quite different from those of single compounds, due to complex complementary, additive or synergistic interactions between polyphenols and/or other classes of phytochemicals.

A growing body of evidence in the literature suggests a link between intestinal dysbiosis and a variety of illnesses and their courses (42, 81), including COVID-19 disease severity (82, 83). The gut microbiome and its composition is highly important for human organism not only due to its participation in digestion, absorption and metabolism, but also due to its modulating activity in the immune responses. Some strains of commensal bacteria species such as *Escherichia coli* and Enterococcus spp. are involved in the production of antibodies, maturation of B lymphocytes and maintaining the balance of Th1/Th2 lymphocytes by activating the cytokine network. Enterococcus bacteria, stimulate plasmocytes in the intestinal epithelium to synthesize secretory IgA, and non-pathogenic *Escherichia coli* strains activate gut-associated lymphoid tissue (GALT) cells to synthesize antimicrobial factors and mature dendritic cells (84).

Polyphenols and phytosterols are among the dietary components suggested in the literature to help maintain intestinal homeostasis (6, 7). Absorbed polyphenols interact with the immune system from the gastrointestinal tract and thus contribute to the prevention of some immune diseases (42, 46). Immune cells express polyphenol receptors enabling the activation of signaling pathways to initiate an immune response (46). It has been documented that SARS-CoV-2-induced dysbiosis of the gut microbiota might be modified by the prebiotic effects of polyphenols (85).

*In vitro* and *in vivo* animal as well as human studies have shown that an adequate diet rich in phytochemicals can promote the growth of beneficial microflora in the gut and suppress pathogenic bacteria (86). Considering the symbiotic relationship of *Escherichia coli* and Enterococcus spp. with the host’s immune system we checked the aforementioned hypotheses for these microbes. In our study we observed a positive association of β-sitosterol intake and phytosterols in total with the occurrence of normal amounts of *Escherichia coli* in stool samples. Additionally, there was a trend of more frequent presence of Enterococcus spp. at the relevant level, i.e., >10^6^ CFU/g, in people with a higher intake of lariciresinol.

These findings are in agreement with the results of other studies. Lariciresinol isolated from Rubia philippinensis showed a reduction in bacterial cell viability and had antimicrobial activity against pathogenic strains of *Escherichia coli* (87). Feeding animals with high doses of stigmasterol (88) or β-sitosterol (48) resulted in the alleviation of intestinal dysbiosis. The consequence was increased cholesterol and coprostanol excretion, as well as decreased hepatic esterified cholesterol (7). Consumption of polyphenols from red wine and dealcoholized red wine significantly increased the number of Enterococcus groups and the concentration of Enterococcus in feces (89, 90). Since dysbiosis of the intestinal microflora is associated with the development of many noncommunicable diseases, including cardiovascular disease, obesity and neurodegenerative diseases, a beneficial interaction with polyphenolic compounds could potentially provide health benefits. With that said, the ability of dietary polyphenols to produce clinical effects may attribute, at least in part, to a bidirectional relationship with the gut microbiota. Polyphenols can influence the composition of the gut microbiota and gut bacteria metabolize polyphenols into bioactive compounds that confer clinical benefits (61).

Although we have made efforts to minimize the probability of bias during conducting this research, our study is not free from certain limitations. Firstly, as it is common in observational studies, we cannot rule out the possibility of residual confounding by unmeasured dietary variables or other factors. Notwithstanding, for counteracting such a threat, the study participation was constrained by some exclusion criteria and we adjusted the intake of all phytochemicals to total energy intake. At the same time, we conducted several types of analyses to check the stability of the results, controlling constructed models to different sets of covariates. Secondly, some lignans, especially matairesinol, were consumed by participants in small amounts and, therefore, the results for them should be interpreted with caution. Thirdly, e.g., due to seasonal variation in the accessibility of fresh fruits and vegetables in Poland, participants could change their dietary patterns during follow-up and might not maintain their eating habits recorded in diaries. However, since a pandemic wave came around the time of the examination, the time of collecting information about dietary practices seems to be optimal to test whether the nutritional status of the body could shape to a great extent the immune responses against SARS-CoV-2. Finally, mostly due to difficulties associated with escalating lockdown restrictions, and consequently shortening duration of the second arm of the study, the final distribution of sex was in favor of men. Additionally, the online recruitment may have led to over- or under-representation of the target population.

Even though COVID-19 vaccines are the main course of action to curb the development of the SARS-CoV-2 pandemic, additional efforts are being made to mitigate the pathological effects of COVID-19 and other viral respiratory diseases.

The antiviral effects of phytochemicals, combined with well-established antioxidant, anti-inflammatory and anti-cholesterol activities, have proven to be effective in the prevention and treatment of COVID-19 and may provide an alternative or adjuvant solution to drug treatment. Especially since they show comparable effects and fewer side effects than pharmaceutical preparations.

Despite possible drug interactions, increasing the supply of phytosterols and polyphenols including lignans in the diet appears to be one of the simplest and safest methods of counteracting the infection and supporting the treatment of COVID-19. In patients infected with SARS-CoV-2, the use of an appropriate dietary model may prove more effective than the use of single purified compounds including dietary supplements.

## 5. Conclusion

The results of the current study support the hypothesis that a diet rich in phytosterols and polyphenols, including lignans, can help to reduce the risk of COVID-19 contraction. Furthermore, the findings suggest that high consumption of their several representatives, namely β-sitosterol and lariciresinol, may be positively associated with the presence at a relevant level of some strains of commensal bacteria species such as *Escherichia coli* and Enterococcus spp. which support immune system. Various phytochemicals can have a differential effect on gut microbiome and influenza-like diseases. Therefore, further research is needed to explore these outcomes in relation to the bioavailability of specific phytosterols and lignans.

## Data availability statement

The original contributions presented in the study are included in the article/Supplementary material, further inquiries can be directed to the corresponding author.

## Ethics statement

The studies involving human participants were reviewed and approved by the Bioethics Committee of Jagiellonian University No. 1072.6120.5.2020 and 1072.6120.202.2019. The patients/participants provided their written informed consent to participate in this study.

## Author contributions

AM, PJ, IB, and JG contributed to conception and design of the study. AM, IB, and JG wrote the first draft of the manuscript. AM performed the statistical analysis. PJ, AW, and AMW investigation. AM, IB, PJ, KK, AW, AMW, JP, and JG wrote sections of the manuscript. All authors contributed to manuscript revision, read, and approved the submitted version.

## Funding

The study was carried out as part of the project supported by the National Science Centre in Poland: “MINIATURA 3” (No. 2019/03/X/NZ9/01550 to PJ) and conducting of the study and its publication was also supported by the Jagiellonian University statutory resources (N43/DBS/000218 to AM).

## Conflict of interest

The authors declare that the research was conducted in the absence of any commercial or financial relationships that could be construed as a potential conflict of interest.

## Publisher’s note

All claims expressed in this article are solely those of the authors and do not necessarily represent those of their affiliated organizations, or those of the publisher, the editors and the reviewers. Any product that may be evaluated in this article, or claim that may be made by its manufacturer, is not guaranteed or endorsed by the publisher.

## References

[1] Ebrahimzadeh-Attari V Panahi G Hebert JR Ostadrahimi A Saghafi-Asl M Lotfi-Yaghin N. Nutritional approach for increasing public health during pandemic of COVID-19: a comprehensive review of antiviral nutrients and nutraceuticals. Health Promot Perspect. (2021) 11:119–36. doi: 10.34172/hpp.2021.17, PMID: 34195036 PMC8233676

[2] Grosso G Godos J Lamuela-Raventos R Ray S Micek A Pajak A. A comprehensive meta-analysis on dietary flavonoid and lignan intake and cancer risk: level of evidence and limitations. Mol Nutr Food Res. (2017) 61:930. doi: 10.1002/mnfr.201600930 27943649

[3] Micek A Godos J Brzostek T Gniadek A Favari C Mena P. Dietary phytoestrogens and biomarkers of their intake in relation to cancer survival and recurrence: a comprehensive systematic review with meta-analysis. Nutr Rev. (2021) 79:42–65. doi: 10.1093/nutrit/nuaa043 32632445

[4] Grosso G Micek A Godos J Pajak A Sciacca S Galvano F. Dietary flavonoid and lignan intake and mortality in prospective cohort studies: systematic review and dose-response meta-analysis. Am J Epidemiol. (2017) 185:1304–16. doi: 10.1093/aje/kww207 28472215

[5] Godos J Vitale M Micek A Ray S Martini D Del Rio D. Dietary polyphenol intake, blood pressure, and hypertension: a systematic review and meta-analysis of observational studies. Antioxidants (Basel). (2019) 8:152. doi: 10.3390/antiox8060152 31159186 PMC6616647

[6] Shakoor H Feehan J Apostolopoulos V Platat C Al Dhaheri AS Ali HI. Immunomodulatory effects of dietary polyphenols. Nutrients. (2021) 13:728. doi: 10.3390/nu13030728 33668814 PMC7996139

[7] Vezza T Canet F de Marañón AM Bañuls C Rocha M Víctor VM. Phytosterols: nutritional health players in the management of obesity and its related disorders. Antioxidants (Basel). (2020) 9:266. doi: 10.3390/antiox9121266 33322742 PMC7763348

[8] Yan B Chu H Yang D Sze K-H Lai P-M Yuan S. Characterization of the lipidomic profile of human coronavirus-infected cells: implications for lipid metabolism Remodeling upon coronavirus replication. Viruses. (2019) 11:73. doi: 10.3390/v11010073 30654597 PMC6357182

[9] Dai X-J Tan L Ren L Shao Y Tao W Wang Y. COVID-19 risk appears to vary across different alcoholic beverages. Front Nutr. (2021) 8:772700. doi: 10.3389/fnut.2021.772700 35047542 PMC8761797

[10] Name JJ Souza ACR Vasconcelos AR Prado PS Pereira CPM. Zinc, vitamin D and vitamin C: perspectives for COVID-19 with a focus on physical tissue barrier integrity. Front Nutr. (2020) 7:606398. doi: 10.3389/fnut.2020.606398 33365326 PMC7750357

[11] Jagielski P Łuszczki E Wnęk D Micek A Bolesławska I Piórecka B. Associations of nutritional behavior and gut microbiota with the risk of COVID-19 in healthy young adults in Poland. Nutrients. (2022) 14:350. doi: 10.3390/nu14020350 35057534 PMC8779092

[12] Witkowska AM Waśkiewicz A Zujko ME Szcześniewska D Stepaniak U Pająk A. Are total and individual dietary lignans related to cardiovascular disease and its risk factors in postmenopausal women? A nationwide study. Nutrients. (2018) 10:865. doi: 10.3390/nu10070865 29973570 PMC6073341

[13] Waśkiewicz A Zujko ME Szcześniewska D Tykarski A Kwaśniewska M Drygas W. Polyphenols and dietary antioxidant potential, and their relationship with arterial hypertension: a cross-sectional study of the adult population in Poland (WOBASZ II). Adv Clin Exp Med. (2019) 28:797–806. doi: 10.17219/acem/91487 30968608

[14] Witkowska AM Waśkiewicz A Zujko ME Mirończuk-Chodakowska I Cicha-Mikołajczyk A Drygas W. Assessment of plant sterols in the diet of adult polish population with the use of a newly developed database. Nutrients. (2021) 13:2722. doi: 10.3390/nu13082722 34444882 PMC8398305

[15] Willett WC Howe GR Kushi LH. Adjustment for total energy intake in epidemiologic studies. Am J Clin Nutr. (1997) 65:1220S–8S; discussion 1229S. doi: 10.1093/ajcn/65.4.1220S 9094926

[16] Gallagher D Heymsfield SB Heo M Jebb SA Murgatroyd PR Sakamoto Y. Healthy percentage body fat ranges: an approach for developing guidelines based on body mass index. Am J Clin Nutr. (2000) 72:694–701. doi: 10.1093/ajcn/72.3.694 10966886

[17] Jagielski P Wnęk D Łuszczki E Bolesławska I Micek A Kozioł-Kozakowska A. Proposition of a new POLA index to assess the immunomodulatory properties of the diet and its relationship with the gut microbiota, using the example of the incidence of COVID-19 in a Group of People without comorbidities. Nutrients. (2022) 14:4227. doi: 10.3390/nu14204227 36296911 PMC9607188

[18] Annunziata G Sanduzzi Zamparelli M Santoro C Ciampaglia R Stornaiuolo M Tenore GC. May polyphenols have a role against coronavirus infection? An overview of in vitro evidence. Front Med (Lausanne). (2020) 7:240. doi: 10.3389/fmed.2020.00240 32574331 PMC7243156

[19] Cai W Wu L-R Zhang S-L. Lignans from Mosla scabra ameliorated influenza a virus-induced pneumonia via inhibiting macrophage activation. Evid Based Complement Alternat Med. (2022) 2022:1688826. doi: 10.1155/2022/1688826 35942373 PMC9356792

[20] Shokry S Hegazy A Abbas AM Mostafa I Eissa IH Metwaly AM. Phytoestrogen β-sitosterol exhibits potent *in vitro* antiviral activity against influenza a viruses. Vaccines (Basel). (2023) 11:228. doi: 10.3390/vaccines11020228 36851106 PMC9964242

[21] Barre A Van Damme EJM Simplicien M Le Poder S Klonjkowski B Benoist H. Man-specific lectins from plants, fungi, algae and cyanobacteria, as potential blockers for SARS-CoV, MERS-CoV and SARS-CoV-2 (COVID-19) coronaviruses: biomedical perspectives. Cells. (2021) 10:1619. doi: 10.3390/cells10071619 34203435 PMC8305077

[22] Gu W Zhao Y Yang L Du M Li Q Ren Z. A new perspective to improve the treatment of Lianhuaqingwen on COVID-19 and prevent the environmental health risk of medication. Environ Sci Pollut Res Int. (2022) 29:74208–24. doi: 10.1007/s11356-022-21125-w 35635661 PMC9148946

[23] Wu Y Pegan SD Crich D Desrochers E Starling EB Hansen MC. Polyphenols as alternative treatments of COVID-19. Comput Struct Biotechnol J. (2021) 19:5371–80. doi: 10.1016/j.csbj.2021.09.022 34567475 PMC8452152

[24] Zhuang Z Zhong X Zhang H Chen H Huang B Lin D. Exploring the potential mechanism of Shufeng Jiedu capsule for treating COVID-19 by comprehensive network pharmacological approaches and molecular docking validation. Comb Chem High Throughput Screen. (2021) 24:1377–94. doi: 10.2174/1386207323999201029122301 33135607

[25] Chojnacka K Witek-Krowiak A Skrzypczak D Mikula K Młynarz P. Phytochemicals containing biologically active polyphenols as an effective agent against COVID-19-inducing coronavirus. J Funct Foods. (2020) 73:104146. doi: 10.1016/j.jff.2020.104146 32834835 PMC7392194

[26] Ngwa W Kumar R Thompson D Lyerly W Moore R Reid T-E. Potential of flavonoid-inspired phytomedicines against COVID-19. Molecules. (2020) 25:2707. doi: 10.3390/molecules25112707 32545268 PMC7321405

[27] Allam AE Amen Y Ashour A Assaf HK Hassan HA Abdel-Rahman IM. *In silico* study of natural compounds from sesame against COVID-19 by targeting Mpro, PLpro and RdRp. RSC Adv. (2021) 11:22398–408. doi: 10.1039/d1ra03937g 35480825 PMC9034212

[28] Paraiso IL Revel JS Stevens JF. Potential use of polyphenols in the battle against COVID-19. Curr Opin Food Sci. (2020) 32:149–55. doi: 10.1016/j.cofs.2020.08.004 32923374 PMC7480644

[29] Ubani A Agwom F Shehu NY Luka P Umera EA Umar U. Molecular docking analysis of some phytochemicals on two SARS-CoV-2 targets. BioRxiv. (2020). doi: 10.1101/2020.03.31.017657

[30] Utomo RY Ikawati M Meiyanto E. Revealing the potency of citrus and galangal constituents to halt SARS-CoV-2 infection. Biology. (2020). doi: 10.20944/preprints202003.0214.v1 [Epub ahead of print].

[31] Mohajeri M Mohajery R Nemati A Pourfarzi F. The difference in the dietary inflammatory index, functional food, and antioxidants intake between COVID -19 patients and healthy persons. Med J Nutrition Metab. (2022) 15:219–27. doi: 10.3233/MNM-211521

[32] Van Laren A Drießen M Rasa S Massar K Ten Hoor GA. Nutritional changes during the COVID-19 pandemic: a rapid scoping review on the impact of psychological factors. Int J Food Sci Nutr. (2023) 74:124–87. doi: 10.1080/09637486.2023.2180613 36823035

[33] Bonaccio M Gianfagna F Stival C Amerio A Bosetti C Cavalieri d’Oro L. Changes in a Mediterranean lifestyle during the COVID-19 pandemic among elderly Italians: an analysis of gender and socioeconomic inequalities in the “LOST in Lombardia” study. Int J Food Sci Nutr. (2022) 73:683–92. doi: 10.1080/09637486.2022.2040009 35285380

[34] Akkuş ÖÖ Atalay BG Parlak E. COVID-19 pandemic: changes in the emotions, body weights and nutrition habits of individuals during social intervention measures. Med J Nutrition Metab. (2022) 15:11–21. doi: 10.3233/MNM-210006

[35] Khan Z Nath N Rauf A Emran TB Mitra S Islam F. Multifunctional roles and pharmacological potential of β-sitosterol: emerging evidence toward clinical applications. Chem Biol Interact. (2022) 365:110117. doi: 10.1016/j.cbi.2022.110117 35995256

[36] Mustafa AM Abouelenein D Acquaticci L Alessandroni L Angeloni S Borsetta G. Polyphenols, saponins and phytosterols in lentils and their health benefits: an overview. Pharmaceuticals (Basel). (2022) 15:1225. doi: 10.3390/ph15101225 36297337 PMC9609092

[37] Cherian A Vadivel V Thiruganasambandham S Madhavankutty S. Phytocompounds and their molecular targets in immunomodulation: a review. J Basic Clin Physiol Pharmacol. (2021). doi: 10.1515/jbcpp-2021-0172 [Epub ahead of print]. 34786892

[38] Brito AF Zang Y. A review of lignan metabolism, milk enterolactone concentration, and antioxidant status of dairy cows fed flaxseed. Molecules. (2018) 24:41. doi: 10.3390/molecules24010041 30583523 PMC6337492

[39] Giovinazzo G Gerardi C Uberti-Foppa C Lopalco L. Can natural polyphenols help in reducing cytokine storm in COVID-19 patients? Molecules. (2020) 25:5888. doi: 10.3390/molecules25245888 33322757 PMC7763290

[40] López-García G Cilla A Barberá R Alegría A. Anti-inflammatory and cytoprotective effect of plant sterol and Galactooligosaccharides-enriched beverages in Caco-2 cells. J Agric Food Chem. (2020) 68:1862–70. doi: 10.1021/acs.jafc.9b03025 31290324

[41] Zhang S Cheng M Wang Z Liu Y Ren Y Rong S. Secoisolariciresinol Diglucoside exerts anti-inflammatory and antiapoptotic effects through inhibiting the Akt/IκB/NF-κB pathway on human umbilical vein endothelial cells. Mediat Inflamm. (2020) 2020:3621261. doi: 10.1155/2020/3621261 PMC7333043 32684834

[42] Magrone T Jirillo E. The interplay between the gut immune system and microbiota in health and disease: nutraceutical intervention for restoring intestinal homeostasis. Curr Pharm Des. (2013) 19:1329–42. doi: 10.2174/138161213804805793 23151182

[43] Mileo AM Nisticò P Miccadei S. Polyphenols: immunomodulatory and therapeutic implication in colorectal cancer. Front Immunol. (2019) 10:729. doi: 10.3389/fimmu.2019.00729 31031748 PMC6470258

[44] Salehi B Quispe C Sharifi-Rad J Cruz-Martins N Nigam M Mishra AP. Phytosterols: from preclinical evidence to potential clinical applications. Front Pharmacol. (2020) 11:599959. doi: 10.3389/fphar.2020.599959 33519459 PMC7841260

[45] Wu Q Wang Y Li Q. Matairesinol exerts anti-inflammatory and antioxidant effects in sepsis-mediated brain injury by repressing the MAPK and NF-κB pathways through up-regulating AMPK. Aging (Albany NY). (2021) 13:23780–95. doi: 10.18632/aging.203649 34705665 PMC8580336

[46] Ding S Jiang H Fang J. Regulation of immune function by polyphenols. J Immunol Res. (2018) 2018:1264074. doi: 10.1155/2018/1264074 29850614 PMC5925142

[47] Yuan L Zhang F Shen M Jia S Xie J. Phytosterols suppress phagocytosis and inhibit inflammatory mediators via ERK pathway on LPS-triggered inflammatory responses in RAW264.7 macrophages and the correlation with their structure. Foods. (2019) 8:582. doi: 10.3390/foods8110582 31744147 PMC6915509

[48] Wu W Liu W Wang H Wang W Chu W Jin J. β-Sitosterol inhibits trimethylamine production by regulating the gut microbiota and attenuates atherosclerosis in ApoE−/− mice. Front Cardiovasc Med. (2022) 9:986905. doi: 10.3389/fcvm.2022.986905 36386330 PMC9663806

[49] Palacios-Rápalo SN De Jesús-González LA Cordero-Rivera CD Farfan-Morales CN Osuna-Ramos JF Martínez-Mier G. Cholesterol-rich lipid rafts as platforms for SARS-CoV-2 entry. Front Immunol. (2021) 12:796855. doi: 10.3389/fimmu.2021.796855 34975904 PMC8719300

[50] Yanagisawa R He C Asai A Hellwig M Henle T Toda M. The impacts of cholesterol, oxysterols, and cholesterol lowering dietary compounds on the immune system. Int J Mol Sci. (2022) 23:12236. doi: 10.3390/ijms232012236 36293093 PMC9602982

[51] Li X Xin Y Mo Y Marozik P He T Guo H. The bioavailability and biological activities of phytosterols as modulators of cholesterol metabolism. Molecules. (2022):27. doi: 10.3390/molecules27020523 35056839 PMC8781140

[52] Plat J Nichols JA Mensink RP. Plant sterols and stanols: effects on mixed micellar composition and LXR (target gene) activation. J Lipid Res. (2005) 46:2468–76. doi: 10.1194/jlr.M500272-JLR200 16150823

[53] Plösch T Kruit JK Bloks VW Huijkman NCA Havinga R Duchateau GSMJE. Reduction of cholesterol absorption by dietary plant sterols and stanols in mice is independent of the Abcg5/8 transporter. J Nutr. (2006) 136:2135–40. doi: 10.1093/jn/136.8.2135 16857831

[54] Yang C McDonald JG Patel A Zhang Y Umetani M Xu F. Sterol intermediates from cholesterol biosynthetic pathway as liver X receptor ligands. J Biol Chem. (2006) 281:27816–26. doi: 10.1074/jbc.M603781200 16857673

[55] Yang C Yu L Li W Xu F Cohen JC Hobbs HH. Disruption of cholesterol homeostasis by plant sterols. J Clin Invest. (2004) 114:813–22. doi: 10.1172/JCI22186 15372105 PMC516266

[56] Gliozzi M Walker R Muscoli S Vitale C Gratteri S Carresi C. Bergamot polyphenolic fraction enhances rosuvastatin-induced effect on LDL-cholesterol, LOX-1 expression and protein kinase B phosphorylation in patients with hyperlipidemia. Int J Cardiol. (2013) 170:140–5. doi: 10.1016/j.ijcard.2013.08.125 24239156

[57] Kočar E Režen T Rozman D. Cholesterol, lipoproteins, and COVID-19: basic concepts and clinical applications. Biochim Biophys Acta Mol Cell Biol Lipids. (2021) 1866:158849. doi: 10.1016/j.bbalip.2020.158849 33157278 PMC7610134

[58] Micek A Godos J Del Rio D Galvano F Grosso G. Dietary flavonoids and cardiovascular disease: a comprehensive dose-response meta-analysis. Mol Nutr Food Res. (2021) 65:e2001019. doi: 10.1002/mnfr.202001019 33559970

[59] Grosso G Godos J Currenti W Micek A Falzone L Libra M. The effect of dietary polyphenols on vascular health and hypertension: current evidence and mechanisms of action. Nutrients. (2022) 14:545. doi: 10.3390/nu14030545 35276904 PMC8840535

[60] Cao H Ou J Chen L Zhang Y Szkudelski T Delmas D. Dietary polyphenols and type 2 diabetes: human study and clinical trial. Crit Rev Food Sci Nutr. (2019) 59:3371–9. doi: 10.1080/10408398.2018.1492900 29993262

[61] Fraga CG Croft KD Kennedy DO Tomás-Barberán FA. The effects of polyphenols and other bioactives on human health. Food Funct. (2019) 10:514–28. doi: 10.1039/c8fo01997e 30746536

[62] Zhang Y Balasooriya H Sirisena S Ng K. The effectiveness of dietary polyphenols in obesity management: a systematic review and meta-analysis of human clinical trials. Food Chem. (2023) 404:134668. doi: 10.1016/j.foodchem.2022.134668 36323021

[63] Babu S Jayaraman S. An update on β-sitosterol: a potential herbal nutraceutical for diabetic management. Biomed Pharmacother. (2020) 131:110702. doi: 10.1016/j.biopha.2020.110702 32882583

[64] Merino J Joshi AD Nguyen LH Leeming ER Mazidi M Drew DA. Diet quality and risk and severity of COVID-19: a prospective cohort study. Gut. (2021) 70:2096–104. doi: 10.1136/gutjnl-2021-325353 34489306 PMC8500931

[65] Vu T-HT Rydland KJ Achenbach CJ Van Horn L Cornelis MC. Dietary Behaviors and incident COVID-19 in the UK biobank. Nutrients. (2021) 13:2114. doi: 10.3390/nu13062114 34203027 PMC8234071

[66] Bonaccio M Pounis G Cerletti C Donati MB Iacoviello L de Gaetano G. Mediterranean diet, dietary polyphenols and low grade inflammation: results from the MOLI-SANI study. Br J Clin Pharmacol. (2017) 83:107–13. doi: 10.1111/bcp.12924 26935858 PMC5338145

[67] Bagnato C Perfetto C Labanca F Negrin LC. The Mediterranean diet: healthy and sustainable dietary pattern in the time of Sars-Cov-2. Med J Nutrition Metab. (2021) 14:365–81. doi: 10.3233/MNM-200597

[68] Pounis G Bonaccio M Di Castelnuovo A Costanzo S de Curtis A Persichillo M. Polyphenol intake is associated with low-grade inflammation, using a novel data analysis from the Moli-sani study. Thromb Haemost. (2016) 115:344–52. doi: 10.1160/TH15-06-0487 26355794

[69] Koistinen VM Tuomainen M Lehtinen P Peltola P Auriola S Jonsson K. Side-stream products of malting: a neglected source of phytochemicals. NPJ Sci Food. (2020) 4:21. doi: 10.1038/s41538-020-00081-0 33311514 PMC7733442

[70] Muller R Walker S Brauer J. Does beer contain compounds that might interfere with cholesterol metabolism? J Inst Brew. (2007) 113:102–9. doi: 10.1002/j.2050-0416.2007.tb00263.x

[71] Sanduzzi Zamparelli S Capitelli L Coppola N Venditto C Santoro C Annunziata G. A phase II study on the effect of Taurisolo® administered via AEROsol in hospitalized patients with mild to moderate COVID-19 pneumonia: the TAEROVID-19 study. Cells. (2022) 11:1499. doi: 10.3390/cells11091499 35563805 PMC9101184

[72] Khan SL Siddiqui FA. Beta-sitosterol: as immunostimulant, antioxidant and inhibitor of SARS-CoV-2 spike glycoprotein. Arch Pharmacol Ther. (2020) 2:12–6. doi: 10.33696/Pharmacol.2.014

[73] Sureja DK Shah AP Gajjar ND Jadeja SB Bodiwala KB Dhameliya TM. *In-silico* computational investigations of AntiViral lignan derivatives as potent inhibitors of SARS CoV-2. Chemistry Select. (2022) 7:e202202069. doi: 10.1002/slct.202202069 35942360 PMC9349937

[74] Pellegrini N Valtueña S Ardigò D Brighenti F Franzini L Del Rio D. Intake of the plant lignans matairesinol, secoisolariciresinol, pinoresinol, and lariciresinol in relation to vascular inflammation and endothelial dysfunction in middle age-elderly men and post-menopausal women living in northern Italy. Nutr Metab Cardiovasc Dis. (2010) 20:64–71. doi: 10.1016/j.numecd.2009.02.003 19361969

[75] O’Driscoll M Ribeiro Dos Santos G Wang L Cummings DAT Azman AS Paireau J. Age-specific mortality and immunity patterns of SARS-CoV-2. Nature. (2021) 590:140–5. doi: 10.1038/s41586-020-2918-0 33137809

[76] Alwani M Yassin A Al-Zoubi RM Aboumarzouk OM Nettleship J Kelly D. Sex-based differences in severity and mortality in COVID-19. Rev Med Virol. (2021) 31:e2223. doi: 10.1002/rmv.2223 33646622 PMC8014761

[77] Yu W Rohli KE Yang S Jia P. Impact of obesity on COVID-19 patients. J Diabetes Complicat. (2021) 35:107817. doi: 10.1016/j.jdiacomp.2020.107817 PMC7690270 33358523

[78] Testino G Pellicano R. COVID-19 and alcohol consumption: recommendations in the omicron era. Minerva Gastroenterol (Torino). (2022). doi: 10.23736/S2724-5985.22.03194-1 [Epub ahead of print]. 35511656

[79] Enaru B Socaci S Farcas A Socaciu C Danciu C Stanila A. Novel delivery systems of polyphenols and their potential health benefits. Pharmaceuticals (Basel). (2021) 14:946. doi: 10.3390/ph14100946 34681170 PMC8538464

[80] Stromsnes K Lagzdina R Olaso-Gonzalez G Gimeno-Mallench L Gambini J. Pharmacological properties of polyphenols: bioavailability, mechanisms of action, and biological effects in *in vitro* studies, animal models, and humans. Biomedicine. (2021) 9:1074. doi: 10.3390/biomedicines9081074 PMC8392236 34440278

[81] Vijay A Valdes AM. Role of the gut microbiome in chronic diseases: a narrative review. Eur J Clin Nutr. (2022) 76:489–501. doi: 10.1038/s41430-021-00991-6 34584224 PMC8477631

[82] Venzon M Bernard-Raichon L Klein J Axelrad JE Zhang C Hussey GA. Gut microbiome dysbiosis during COVID-19 is associated with increased risk for bacteremia and microbial translocation. BioRxiv. (2022). doi: 10.1101/2021.07.15.452246 [Epub ahead of print]. PMC9626559 36319618

[83] Bottari B Castellone V Neviani E. Probiotics and COVID-19. Int J Food Sci Nutr. (2021) 72:293–9. doi: 10.1080/09637486.2020.1807475 32787470

[84] Gałecka M Basińska AM Bartnicka A. Znaczenie mikrobioty jelitowej w kształtowaniu zdrowia człowieka—implikacje w praktyce lekarza rodzinnego. Forum Medycyny Rodzinnej. (2018) 12:50–9.

[85] Calcuttawala F. Nutrition as a key to boost immunity against COVID-19. Clin Nutr ESPEN. (2022) 49:17–23. doi: 10.1016/j.clnesp.2022.04.007 35623808 PMC9012504

[86] Wang X Qi Y Zheng H. Dietary polyphenol, gut microbiota, and health benefits. Antioxidants (Basel). (2022) 11:1212. doi: 10.3390/antiox11061212 35740109 PMC9220293

[87] Bajpai VK Shukla S Paek WK Lim J Kumar P Kumar P. Efficacy of (+)-Lariciresinol to control bacterial growth of Staphylococcus aureus and *Escherichia coli* O157:H7. Front Microbiol. (2017) 8:804. doi: 10.3389/fmicb.2017.00804 28515721 PMC5413575

[88] Andriamiarina R Laraki L Pelletier X Debry G. Effects of stigmasterol-supplemented diets on fecal neutral sterols and bile acid excretion in rats. Ann Nutr Metab. (1989) 33:297–303. doi: 10.1159/000177548 2516429

[89] Queipo-Ortuño MI Boto-Ordóñez M Murri M Gomez-Zumaquero JM Clemente-Postigo M Estruch R. Influence of red wine polyphenols and ethanol on the gut microbiota ecology and biochemical biomarkers. Am J Clin Nutr. (2012) 95:1323–34. doi: 10.3945/ajcn.111.027847 22552027

[90] Boto-Ordóñez M Urpi-Sarda M Queipo-Ortuño MI Tulipani S Tinahones FJ Andres-Lacueva C. High levels of Bifidobacteria are associated with increased levels of anthocyanin microbial metabolites: a randomized clinical trial. Food Funct. (2014) 5:1932–8. doi: 10.1039/c4fo00029c 24958563

