# Supplementary material for: Association of dietary intake of polyphenols, lignans, and phytosterols with immune-stimulating microbiota and COVID-19 risk in a group of Polish men and women
Source: Front Nutr. 2023 Aug 3;10:1241016. doi: 10.3389/fnut.2023.1241016 (PMC10436747; doi:10.3389/fnut.2023.1241016)
Supplement: Supplementary file 1 [file Table_1.DOCX]

Supplementary Material

**The association of polyphenol, lignan and phytosterol intake in diet with the immunostimulatory microbiota and the incidence of COVID-19 disease in a group of Polish men and women**

**Agnieszka Micek^*^, Izabela Bolesławska, Paweł Jagielski, Kamil Konopka, Anna Waskiewicz, Anna Maria Witkowska, Juliusz Przysławski, Justyna Godos**

*** Correspondence:** Agnieszka Micek agnieszka.micek@uj.edu.pl

Supplementary Table 1. Comparison of distribution of energy adjusted logarithmically transformed daily consumption of specific phytochemicals between respondents who have contracted and who have not contracted COVID-19 (N=95).

| **Variable** | **Have not contracted COVID-19 (N=71)** | **Have contracted COVID-19 (N=24)** |
| --- | --- | --- |
| **Logarithm^#^ of total polyphenols [mg]** | 11.36 (0.42) | 11.15 (0.39)* |
| **Logarithm^#^ of total lignans [μg]** | 5.24 (1.14) | 4.89 (1.06) |
| **Logarithm^#^ of lariciresinol [μg]** | 4.37 (1.02) | 4.09 (0.92) |
| **Logarithm^#^ of matairesinol [ng]** | 6.71 (1.97) | 6.34 (1.70) |
| **Logarithm^#^ of pinoresinol [μg]** | 3.38 (1.62) | 3.10 (1.65) |
| **Logarithm^#^ of secoisolariciresinol [μg]** | 1.61 (1.44) | 0.57 (1.32)** |
| **Logarithm^#^ of total phytosterols [mg]** | 8.55 (0.51) | 8.29 (0.30)** |
| **Logarithm^#^ of stigmasterols [mg]** | 5.43 (0.86) | 4.86 (0.43)*** |
| **Logarithm^#^ of campesterols [mg]** | 5.98 (0.43) | 5.98 (0.44) |
| **Logarithm^#^ of β-Sitosterols [mg]** | 7.83 (0.50) | 7.59 (0.29)** |

^#^Logarithm with base 2 was used in transformation, data are presented as means (sds), *P<0.05, **P<0.01, ***P<0.001 for Student T-test analysis (N=95).

Supplementary Table 2. Distribution of bacteria Enterococcus spp. and Escherichia coli outside the norm with analysis of trend across specific-phytochemical tertiles (N=95).

| **Pohytochemicals** | **Phytochemical-intake** | | | **Phytochemical intake** | | | **Per 1 category of tertile** |
| --- | --- | --- | --- | --- | --- | --- | --- |
|  | **T1** | **T2** | **T3** | **T1** | **T2** | **T3** | **increase** |
|  |  | **n (%)** |  |  | **OR^&^ (95% CI)** |  | **OR^&^ (95% CI)** |
| ***Enterococcus spp.*** |  |  |  |  |  |  |  |
| **Total polyphenols** | 22 (68.8) | 19 (61.3) | 18 (56.3) | 1 (ref.) | 0.67 (0.23-1.93) | 0.57 (0.19-1.65) | 0.77 (0.46-1.27) |
| **Total lignans** | 23 (71.9) | 20 (64.5) | 16 (50.0) | 1 (ref.) | 0.71 (0.24-2.13) | 0.40 (0.13-1.13) | 0.62 (0.37-1.05) |
| **Lariciresinols** | 24 (75.0) | 19 (61.3) | 16 (50.0) | 1 (ref.) | 0.52 (0.17-1.53) | 0.34 (0.11-0.96)* | 0.58 (0.34-0.98)* |
| **Matairesinols** | 23 (71.9) | 19 (61.3) | 17 (53.1) | 1 (ref.) | 0.65 (0.22-1.90) | 0.42 (0.14-1.22) | 0.67 (0.40-1.12) |
| **Pinoresinols** | 22 (68.8) | 19 (61.3) | 18 (56.3) | 1 (ref.) | 0.70 (0.23-2.07) | 0.59 (0.20-1.68) | 0.77 (0.46-1.27) |
| **Secoisolariciresinols** | 21 (65.6) | 21 (67.7) | 17 (53.1) | 1 (ref.) | 1.14 (0.39-3.37) | 0.56 (0.19-1.61) | 0.77 (0.46-1.27) |
| ***Escherichia coli*** |  |  |  |  |  |  |  |
| **Total phytosterols** | 17 (53.1) | 11 (35.5) | 12 (37.5) | 1 (ref.) | 0.55 (0.18-1.59) | 0.34 (0.10-1.08)' | 0.72 (0.44-1.20) |
| **Stigmasterols** | 17 (53.1) | 10 (32.3) | 13 (40.6) | 1 (ref.) | 0.42 (0.14-1.23) | 0.44 (0.13-1.38) | 0.77 (0.47-1.27) |
| **Campesterols** | 15 (46.9) | 12 (38.7) | 13 (40.6) | 1 (ref.) | 0.70 (0.24-2.00) | 0.67 (0.23-1.87) | 0.88 (0.53-1.45) |
| **β-Sitosterols** | 18 (56.3) | 11 (35.5) | 11 (34.4) | 1 (ref.) | 0.39 (0.13-1.11) | 0.28 (0.08-0.86)* | 0.63 (0.38-1.05) |

T1, T2, T3 – tertile groups, *P<0.05, **P<0.01, ***P<0.001 from Chi-squared test of independence or logistic regression test for trend, ^&^adjusted to age, sex, diet and total energy intake.

Supplementary Table 3. Distribution of POLA index assessing the immunomodulatory properties of the diet by tertiles of lignan and phytosterol intake (N=95).

|  | **Total lignan intake** | | | **Total phytosterol intake** | | |
| --- | --- | --- | --- | --- | --- | --- |
|  | **T1 (N=32)** | **T2 (N=31)** | **T3 (N=32)** | **T1 (N=32)** | **T2 (N=31)** | **T3 (N=32)** |
| ***POLA index scores*^&^** |  |  |  |  |  |  |
| ***BIM, N=37*** | 10 (31.3) | 13 (41.9) | 14 (43.8) | 7 (21.9) | 14 (45.2) | 16 (50.0)* |
| ***UBIM, N=28*** | 11 (34.4) | 9 (29.0) | 8 (25.0) | 8 (25.0) | 9 (29.0) | 11 (34.4) |
| ***HUBIM, N=30*** | 11 (34.4) | 9 (29.0) | 10 (31.3) | 17 (53.1) | 8 (25.8) | 5 (15.6) |

Results are expressed as n (%), T1, T2, T3 – tertile groups, ^&^BIM - beneficial immunomodulation, UBIM - unbeneficial immunomodulation, HUBIM - highly unbeneficial immunomodulation, *P<0.05 from Chi-squared test of independence (N=95).
